# Research Progress on Low Damage Grasping of Fruit, Vegetable and Meat Raw Materials

**DOI:** 10.3390/foods12183451

**Published:** 2023-09-15

**Authors:** Zeyu Xu, Wenbo Shi, Dianbo Zhao, Ke Li, Junguang Li, Junyi Dong, Yu Han, Jiansheng Zhao, Yanhong Bai

**Affiliations:** 1 College of Food and Bioengineering, Zhengzhou University of Light Industry, Zhengzhou 450000, China; happyzeyu123@126.com (Z.X.); wenbo836@163.com (W.S.); zhaodb212@163.com (D.Z.); like@zzuli.edu.cn (K.L.); 2014081@zzuli.edu.com (J.L.); dongjy1999@163.com (J.D.); hanyuatt@163.com (Y.H.); 2 Key Laboratory of Cold Chain Food Processing and Safety Control (Zhengzhou University of Light Industry), Ministry of Education, Zhengzhou 450000, China; 3 Henan Key Laboratory of Cold Chain Food Quality and Safety Control, Zhengzhou 450000, China; 4 Henan Shuanghui Investment & Development Co., Ltd., Luohe 462000, China; zjs4567@163.com

**Keywords:** food raw materials, low damage grasping, robot gripper, control strategies

## Abstract

The sorting and processing of food raw materials is an important step in the food production process, and the quality of the sorting operation can directly or indirectly affect the quality of the product. In order to improve production efficiency and reduce damage to food raw materials, some food production enterprises currently use robots for sorting operations of food raw materials. In the process of robot grasping, some food raw materials such as fruits, vegetables and meat have a soft appearance, complex and changeable shape, and are easily damaged by the robot gripper. Therefore, higher requirements have been put forward for robot grippers, and the research and development of robot grippers that can reduce damage to food raw materials and ensure stable grasping has been a major focus. In addition, in order to grasp food raw materials with various shapes and sizes with low damage, a variety of sensors and control strategies are required. Based on this, this paper summarizes the low damage grasp principle and characteristics of electric grippers, pneumatic grippers, vacuum grippers and magnetic grippers used in automated sorting production lines of fruit, vegetable and meat products, as well as gripper design methods to reduce grasp damage. Then, a grasping control strategy based on visual sensors and tactile sensors was introduced. Finally, the challenges and potential future trends faced by food robot grippers were summarized.

## 1. Introduction

**Figure 1:**
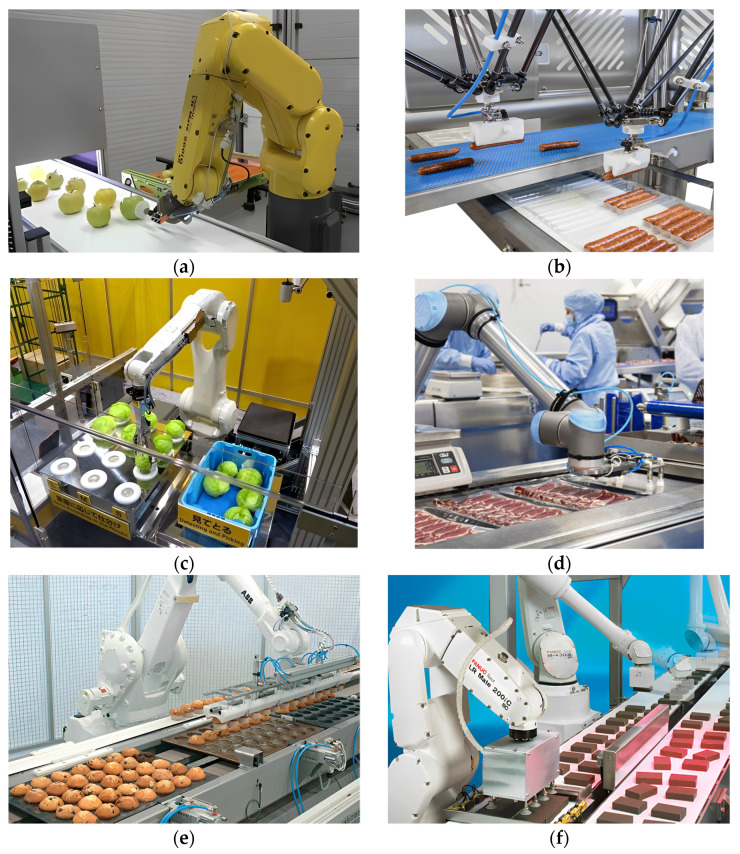
Automated processing production line for sorting robots in food factories: (**a**) The grading and transportation process of fruits; (**b**) the sorting and packaging process of sausages; (**c**) the sorting and packing process of vegetables; (**d**) automated grading and sorting of meat raw materials; (**e**) automated production and transportation process of bread products; (**f**) the production and packaging process of biscuits.

The sorting and processing of food raw materials, as an important link in the production process, is currently mainly completed by manual work, which has problems such as low efficiency, high costs and high labor intensity [1]. In recent years, the gradual increase in labor costs has become a common problem faced by many countries [2]. The demand for using sorting robots to achieve the mechanization and automation of food raw material processing is becoming increasingly urgent. In food production factories, some enterprises have achieved the automated production and processing of sorting robots. Figure 1 shows the automated production line of sorting robots in food factories, and Figure 1a shows the grading and transportation process of fruits in food factories; Figure 1b shows the automated sorting and packaging process of sausages in a food factory; Figure 1c shows the sorting and packing process of vegetables in a food factory; Figure 1d shows the automated grading and sorting of meat raw materials in a food factory; Figure 1e shows the automated production and transportation process of bread products in a food factory; Figure 1f shows the production and packaging process of biscuits in a food factory.

As an important component of sorting robots, the performance of the gripper has a significant impact on product quality [3]. In the non-food industry, the research into and application of robot grippers in structured scenarios are relatively mature. However, in the process of grabbing food raw materials, the complexity of the gripper is much higher than that of structured scene work. Some food raw materials, such as fruits, vegetables, meat, etc., have a soft appearance and complex and variable shapes [4]. Directly using non-food industry sorting robot grippers can easily lead to material damage by the gripper during the sorting process. Therefore, the research into sorting robot grippers is imperative, as their work quality will directly affect the storage and subsequent processing of food raw materials [5]. In addition, although some food companies have already achieved automated sorting, with the development of technology, higher requirements have been put forward for sorting robot grippers and their control strategies. Based on this, the research into and development of robot grippers and control strategies that can reduce damage to food raw materials are of great significance for improving production efficiency, solving manual problems, and ensuring the quality of food raw materials.

At present, robot grippers used for fruit, vegetable and meat raw materials mainly include electric grippers, pneumatic grippers, vacuum grippers and magnetic grippers, which are classified based on their different actuation methods. Fruit, vegetable and meat raw materials generally have characteristics such as easy damage, large volume and heavy weight. It is difficult to achieve low damage while grasping these food raw materials by studying a single type of grasping method. Based on this, this paper summarizes the research into electric grippers, pneumatic grippers, vacuum grippers and magnetic grippers, as well as grasping control strategies for fruit, vegetable and meat raw material sorting robots, and discusses the future development trends in order to provide relevant information and research references for the research of low-damage grasping grippers and control strategies for fruit, vegetable and meat raw materials.

In the collection of paper references, search engines including but not limited to Web of Science, IEEE Xplore, and CNKI were used. The main search keywords were robot gripper, low damage grasping, grasping simulation and grasping control, with a focus on analyzing relevant literature published in the past five years.

## 2. Research Progress of Fruit, Vegetable and Meat Grippers

Based on different usage requirements, different actuation types, and the requirements for work reliability, robot grippers that can be suitable for different working environments are gradually being developed. According to the actuation methods of robot grippers [6], grippers can be classified into four types: electric actuation, pneumatic actuation, vacuum actuation and magnetic actuation. Electric and pneumatic grippers are the most commonly used grippers. Electric grippers can easily and accurately implement control. Pneumatic grippers can provide higher clamping force. The contact vacuum gripper creates viscosity by creating negative pressure at the interface between the sucker and the object, and the suction force is relatively large. A non-contact vacuum gripper operates according to Bernoulli’s principle, creating a high-speed airflow between the gripper and the surface of the object, resulting in a vacuum adsorption grab effect [7]. The magnetic gripper is able to grasp the target object easily by applying a magnetic field to create a form of solidification.

### 2.1. Electric Grippers

**Figure 2:**
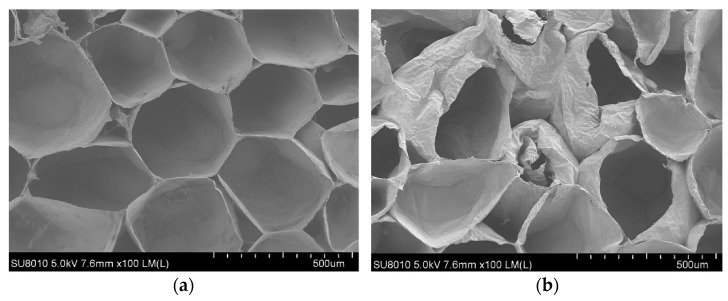
The rupture and deformation of damaged tomato tissue cells under different compression pressures under scanning electron microscopy: (**a**) Healthy cells before tomato damage; (**b**) cell shrinkage after tomato compression injury.

**Figure 3:**
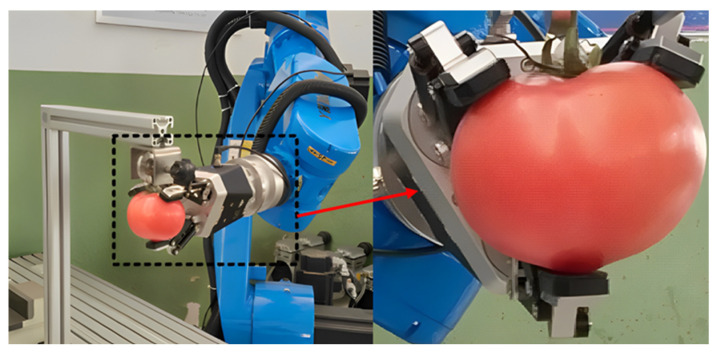
Experimental model diagram of three-finger electric gripper grasping tomato.

Electric grippers can improve their precise grasping ability, and position, acceleration and force sensors can be easily introduced to achieve real-time monitoring and precise control. Electric grippers are the most commonly used grippers, commonly used to grasp easily damaged fruits, vegetables and fragile foods. If a traditional electric gripper does not have any damage reduction measures, it is difficult to avoid causing some mechanical damage to the grasping object. In order to investigate the mechanical damage caused by conventional electric grippers to tomatoes, Wang et al. [8] from Nanjing Agricultural University conducted compression experiments on tomatoes. Through the compression experiments, the influence of local compression pressure on various levels of tomato damage was analyzed, and the epidermal damage and cell deformation under different compression pressures were observed in the macroscopic and microscopic analyses of tomatoes. Therefore, the pressure distribution range of tomato damage levels was obtained. As shown in Figure 2, scanning electron microscopy was used to observe the rupture and deformation of damaged tomato tissue cells under different compression pressures. Figure 2a shows healthy cells before tomato injury, and Figure 2b shows cell shrinkage after tomato compression injury. Finally, based on the pressure distribution intervals of various damage levels in tomatoes, a gripping experiment was conducted using a three-finger electric gripper. The gripping experimental model is shown in Figure 3, and the obtained experimental results are consistent with the evaluation results of mechanical damage caused by local compression in tomatoes.

Therefore, there is still great research space for electric grippers that can achieve low-damage grasping. Because the compliant mechanism or the flexible material on the gripper can adapt to the shape of the grasped object and reduce the damage, there are many studies on the compliant mechanism and flexible material of the electric gripper.

**Figure 4:**
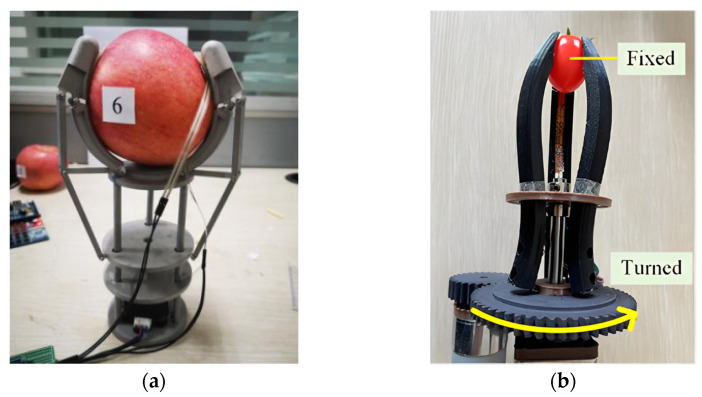
Design of biomimetic grippers based on the shapes of objects: (**a**) Underactuated end-effector for arc-shaped fingers; (**b**) gripper with sinusoidal curve characteristics [12].

Based on the principles of bionics, the gripper is designed according to the shape of the object, such that it can fit with the grasped object during grasping, thereby reducing grasping damage. Li [9], based on the bionics principle and under-actuated principle, combined with the overall dimensions and mechanical properties of the apple, made a specific structural design of the end-effector from the perspective of practicality, as shown in Figure 4a. From the perspective of bionics, the length of a human finger is closely related to the size of the object grasped, so the size of the finger should be determined according to the size of the apple [10]. There are two common types of gripper finger shapes: plane fingers and arc-shaped fingers. During contact with apples, the contact area between arc-shaped fingers and apples is larger than that of plane fingers [11]. Therefore, arc-shaped fingers were used, and flexible materials were applied inside the fingers to improve the adaptive ability of the gripper. Finally, a simulation analysis was conducted to evaluate the changes in contact force between the apple and different knuckles of the fingers, as well as the displacement and velocity changes of the fingers during the grasping process, verifying the adaptive grasping ability and the stability of the under-actuated arc-shaped finger. Due to the formation of a more fixed arc during the grasping process of picking small fruits such as cherry tomatoes and plums, Zhang et al. [12] also designed a gripper with sine curve characteristic fingers based on biomimetic principles, as shown in Figure 4b. The structural parameters of the gripper were determined while meeting the requirements of the grabbing range. A structural model of a single finger of the gripper was established. Based on this model, grasping experiments were conducted at six speeds and with three fruit sizes. The results show that when the speed is 0.08 m/s, the gripping time and maximum gripping force during clamping meet the requirements of high-speed and low-damage sorting.

**Figure 5:**
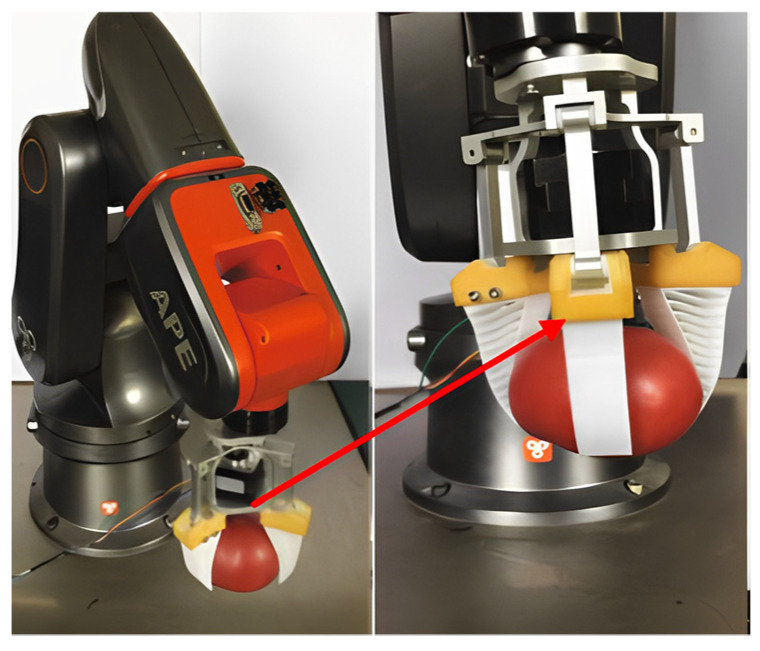
Flexible underactuated end-effector for tomato grasping [14].

**Figure 6:**
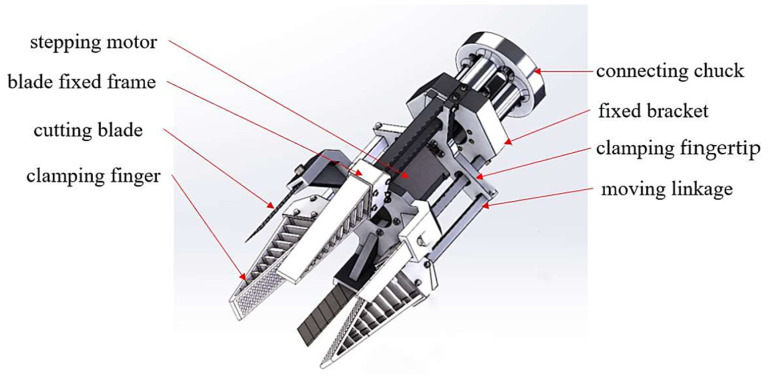
Structural diagram of fin-shaped flexible three finger grasping end-effector [15].

**Figure 7:**
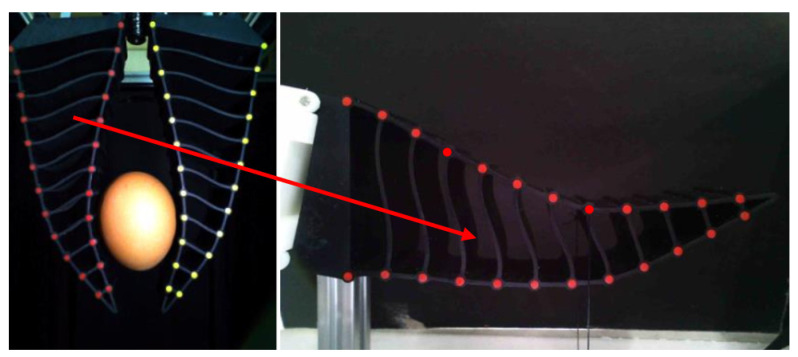
Flexible gripper based on bionics principle.

The Fin Ray Effect (FRE) structure is inspired by the biological structure of fish fins. When one side of the fin is pushed by the hand, the fin does not bend in the direction of the force, but in the opposite direction [13]. The FRE structure can adaptively wrap the object without any external actuation through the passive deformation generated when in contact with the target object. Guo et al. [14] combined the bionic principle of the FRE structure with the design of gripper fingers to design a flexible underactuated end-effector for tomato grasping, as shown in Figure 5. The optimal structural parameters of the fingers were determined through finite element analysis, and tomato grasping experiments were conducted. The results show that the flexible gripper is capable of the non-destructive grasping of tomatoes with a diameter between 65 and 95 mm, and can withstand a tensile force of 7 N, with a load of more than twice its own weight. Xiao et al. [15] designed a fin-shaped flexible three finger grasping end-effector, as shown in Figure 6. Its structure mainly includes: 1. connecting chuck, 2. fixed bracket, 3. clamping fingertip, 4. moving linkage, 5. stepping motor, 6. blade fixed frame, 7. cutting blade, and 8. clamping finger. The end-effector can stably clamp according to the different sizes of fruits, without damaging the skin during the gripping process. The gripper can be 3D-printed with ABS material, resulting in lower manufacturing costs. It has the advantages of strong adaptability, stable grip and causing no damage to the fruit. Zhang [16] from the Harbin Institute of Technology designed a flexible gripper based on bionics to meet the needs of adaptive grasping, as shown in Figure 7, which can adaptively grasp objects of different shapes. The fingers of the flexible gripper were designed using the concept of biomimetic fish fins, with two support plates in the longitudinal direction, one for the contact surface with the object and the other for the back support of the fingers. In order to facilitate the grasping of objects, the support plate of the contact surface must employ an arc design, which can form an initial envelope for the object being grasped when it first comes into contact. The use of flexible materials during manufacturing ensures low damage during grasping. The total mass of this flexible gripper is 838 g, and it can grasp objects with a maximum mass of 2 kg and has excellent load capacity. The gripper designed with a bionic fin structure can be passively deformed when in contact with the target object, and can comply with the wrapped object without any external drive, while ensuring a non-destructive grip and high grip strength.

Liu et al. [17,18] designed and fabricated two different types of robot soft grippers using 3D printing methods. These can adaptively grasp fruits based on their outer diameter and shape contours, and the designed flexible double finger gripper can be actuated by one linear actuator. A monolithic flexible finger was designed by the topology optimization method, and the flexible finger was manufactured by 3D printing using a flexible thermoplastic material. The grasping experiment showed that the flexible gripper can automatically grasp various fruits without causing damage, and can adaptively grasp fragile and irregular objects. These designed grippers have low manufacturing costs, do not require sensors and mechanical joints, achieve a high clamping force, and do not require complex control strategies for clamping.

**Figure 8:**
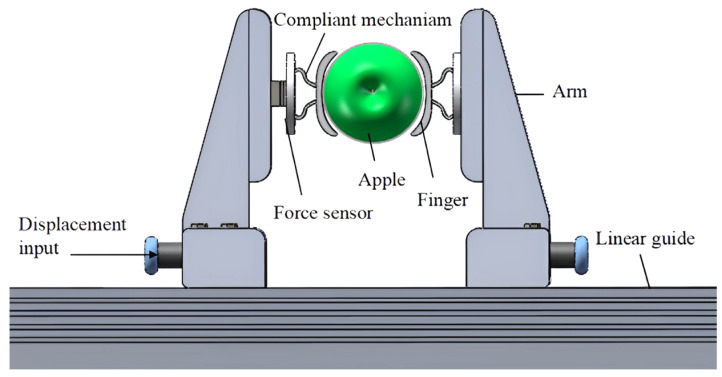
End-effector with compliant mechanism [19].

In order to reduce fruit damage during the clamping process, Miao et al. [19] achieved the constant output force required for low-destructive fruit clamping by setting a compliant mechanism on the end-effector and optimizing the mechanical characteristics of the compliant mechanism, as shown in Figure 8. Firstly, based on the shape function, the nonlinear ordinary differential equation of buckling deformation of flexible beams with boundary conditions was established. Secondly, the above boundary value problem was rephrased as an initial value problem, and the initial value optimization solution was combined with a genetic algorithm. Finally, the shape function of the beam was optimized by the sequential quadratic programming method to achieve a constant force output within a certain deformation range. Taking apple grasping as an example, the initial shape parameters of the compliant mechanism were set, and a constant clamping force of approximately 7.9 N was obtained. The feasibility of this method was verified through nonlinear finite element simulation, force displacement experiments, and apple clamping experiments. 

**Figure 9:**
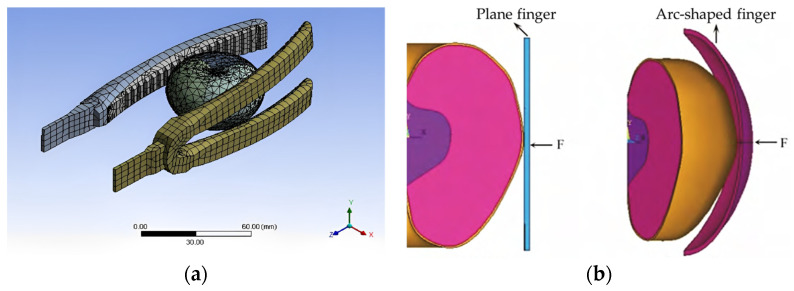
Finite element simulation of grasping process: (**a**) Simulation of grasping tomato with fingers of the gripper; (**b**) simulation models for grasping apples with different types of fingers [21].

The finite element simulation of the grasping process can enable us to conveniently adjust various experimental parameters, reduce the number of actual experiments, save experimental costs, and shorten the development cycle of the gripper. Wang [20] conducted a simulation experiment on tomato picking grippers using ANSYS 19.2 software, and selected the finger material causing the least damage to the fruit. She first used SolidWorks 2020 software to create three-dimensional models of the gripper fingers and tomatoes, using rubber and silicone materials for the surface of the gripper fingers. Then, the model was imported into ANSYS 19.2 software for the grasping simulation, as shown in Figure 9a. The results show that the gripper using silicone as the finger surface material was more suitable for grasping tomato fruits. Finally, a gripper with silicone material as the finger surface was produced and validated through experiments, in which almost no tomatoes were damaged by the gripper. Ji et al. [21] studied the stress changes in the internal tissues of apples when grippers with different finger shapes grasped them, proving that arc-shaped fingers have a lower probability of damaging the apple tissue than plane fingers. He first used ANSYS 19.2 software to establish a finite element model of an apple composed of skin, flesh and core. Then, simulation experiments were conducted on apple grasping using plane- and arc-shaped grippers, as shown in Figure 9b, to obtain Von Mises stress cloud maps of nodes in various parts of the apple. The analysis showed that when the loading force was the same, the stress and deformation caused by the arc-shaped finger in apples were smaller than those caused by the plane finger. Finally, a self-developed two arc-shaped finger gripper was used for validation experiments, and the results show that under the same clamping force, the difference in apple shape variables between the simulation and the experiment did not exceed 10%.

In surgery, low-damage grasping has also been studied. In order to prevent the biological soft tissue from sliding out of the gripper due to the low pinch force during surgery [22], or soft tissue damage due to high pinch forces [23], van Assenbergh et al. [24] developed a modular biological soft tissue gripper. The gripper features an elastic cushion made of carbon fiber fabric, which can enhance the stiffness in its shear direction. This elastic cushion has a lower normal stiffness that maximizes contact with the grasping object without the need for higher pinch forces. Carbon fiber fabrics provide high shear stiffness to ensure that objects can maintain full contact even when shear forces are generated during lifting.

### 2.2. Pneumatic Grippers

**Figure 10:**
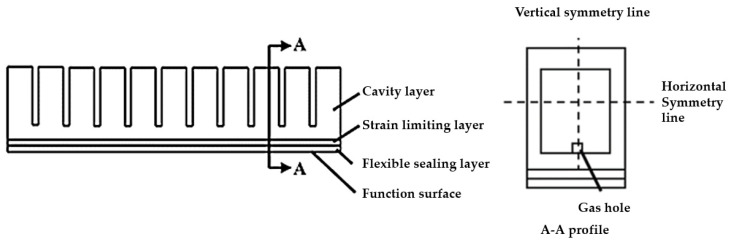
Structure diagram of pneumatic gripper.

The pneumatic gripper can perform a unilateral bending motion in two-dimensional space, and its bending degree is determined by its own structure and the pressure inside the cavity. The structure of the pneumatic gripper is shown in Figure 10, with the main body being a linear array arranged in a tubular structure made of silicone rubber, which is mainly composed of four parts: cavity layer, strain limiting layer, flexible sealing layer with functional surface, and gas hole [25]. The gas hole serves as a gas connection channel, ensuring a good connection between the power source and the interior of the cavity. The cavity serves as a space for storing power sources, providing conditions for the deformation of the pneumatic gripper. The strain limiting layer is mainly used to limit the overall axial motion of the flexible gripper, transforming the axial motion into radial bending. The flexible sealing layer provides the assurance of overall airtightness. Finally, the gas hole adheres to the trachea, and the power source enters the interior of the pneumatic gripper chamber. 

**Figure 11:**
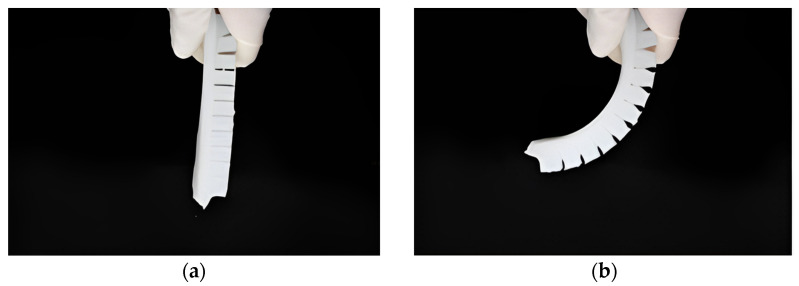
Soft pneumatic gripper finger bending shape under different air pressures: (**a**) Finger shape without air pressure; (**b**) finger shape with increased pressure.

When the pneumatic gripper is working, the interior of the cavity and the gas power source are connected through a gas hole. When the gas fills the interior of the cavity, the cavity expands to squeeze each side, and the whole piece undergoes axial elongation. However, due to the strain limiting layer, the axial elongation of the soft gripper is limited, transforming the overall axial elongation into radial bending. As shown in Figure 11a, when there is no pressure in the cavity, the fingers of the soft gripper do not show significant bending. As shown in Figure 11b, as the pressure in the cavity increases, the fingers of the soft gripper show significant radial bending.

Pneumatic grippers are widely used due to their high robustness, high gripping speed and grip strength, low cost and ease of control [26,27]. Recently, researchers have conducted extensive research on pneumatic grippers. At the National University of Singapore, Low et al. [28] developed a pneumatic soft gripper. It consists of four pneumatic finger actuators, a shell with through holes, and an adjustable base. The grasping length and width can be configured easily to suit a variety of objects. It is capable of picking up and holding objects with a variety of contours, from small, light objects weighing less than 50 g to larger objects weighing 1100 g. Traditional pneumatic finger actuators require the following key steps during production, such as the 3D printing of molds, casting, demolding, and the sealing of pneumatic channel features. Compared to traditional production methods, the production of the entire pneumatic finger actuator was directly completed using a flexible 3D printing filament. This method imparts upon the pneumatic finger actuators better airtightness, compressive strain, and bending ability. 

Wang et al. [29] designed and manufactured a soft pneumatic actuator using multi material 3D printing technology. In order to obtain the maximum deformation under given air pressure conditions, he studied the structural characteristics of single-material actuators and dual-material actuators, obtained the optimal actuator design, and verified it through experimental data on one-degree bending. Finally, using the optimal actuator design, a three-finger soft gripper was developed using a dual material actuator. This gripper is capable of grasping objects of various sizes and shapes, with good gripping performance and adaptability. Salem et al. [30] designed a similar soft pneumatic gripper, consisting of four soft bending actuators and a soft wrist. The pneumatic actuator of this soft gripper was manufactured using 3D printing technology and two different types of TPU 3D printing materials. This soft pneumatic gripper is prone to deformation and can form a suitable shape for grasping objects regardless of the shape of the target object. Compared to Low et al. using a soft printing material for the production of pneumatic finger actuators, they used multi-material 3D printing technology to make pneumatic finger actuators more stable in performance. When printing the chamber, a soft material similar to rubber is used, while a harder material is used at the bottom of the pneumatic finger actuator to prevent damage due to excessive expansion and generate sufficient clamping force.

**Figure 12:**
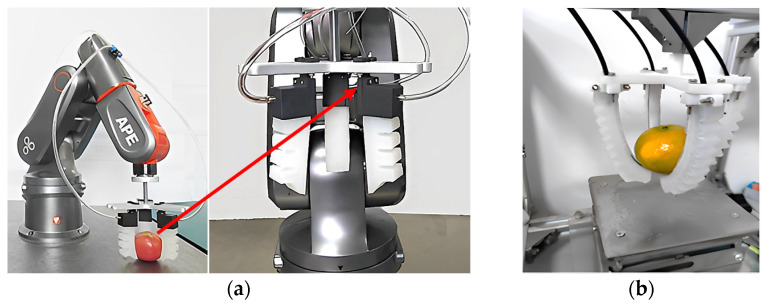
Pneumatic gripper designed through simulation optimization of fingers: (**a**) Gas elastic-driven sorting end-effector based on simulation optimization; (**b**) 3D printing technology pneumatic soft gripper based on simulation optimization [32].

Zhou from Jiangsu University [31] designed a sorting end-effector based on gas elastic drive through Ansys Workbench finite element simulation and genetic algorithm optimization, as shown in Figure 12a. Due to the anisotropy of the finger structure of the end-effector, after pressurizing the finger cavity, the finger can bend towards the bottom and generate clamping force. By simulating the clamping of flexible fingers, the effects of the number of finger chambers, the contour size of the grasped object and the driving pressure on the total clamping force, horizontal clamping force and vertical clamping force were studied. Based on the simulation results of radial and axial clamping forces, as well as the design objectives of optimizing driving cost and manufacturing cost, the optimal number of chambers was four, the optimal hand index was 3, and the optimal driving pressure was 31.645 kPa. The optimized sorting end-effector can perform the non-destructive sorting of tomatoes at a pressure of 32 kPa. Lei et al. [32] conducted simulation analyses on the finger bending deformation of a soft gripper under changes in input air pressure, established a bending deformation model of the finger air chamber, and simulated the motion of the soft gripper using ABAQUS 2020 finite element software. Through simulation, the optimal structural parameters for preparing the soft gripper were determined. Using 3D printing technology, the preparation of a pneumatic soft gripper was completed, and the prepared pneumatic soft gripper is shown in Figure 12b. The theoretical simulation and grasping test results indicate that the soft gripper can grasp objects of different shapes without destructive effects. They all carried out finite element simulations on the soft fingers of sorting gripper, analyzed the bending performance and clamping performance of soft fingers, and completed the preparation of a pneumatic gripper.

**Figure 13:**
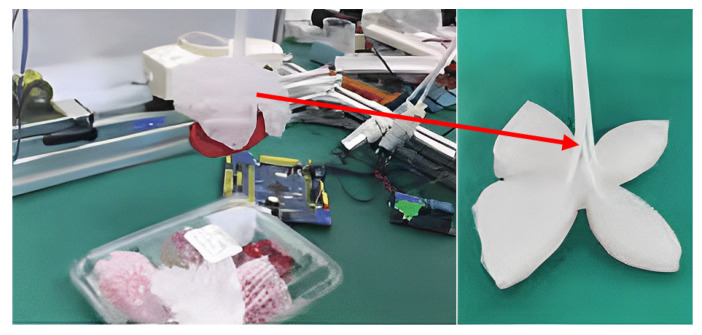
Pneumatic four-piece soft gripper designed with strawberry contour curve.

Currently, most pneumatic grippers have not been subjected to profiling design. Li et al. [33] used the external contour curve of strawberries as a profiling design curve and designed a novel type of pneumatic four-blade soft gripper, as shown in Figure 13. Firstly, the structure of the soft gripper was simulated and optimized, and a grasping method of safely attaching to the surface of the object was proposed. Further, after conducting the minimum failure stress test on the surfaces of strawberries, the safe grasping force of the soft gripper was obtained, and the feasibility of non-destructive grasping using the gripper was verified. Finally, the bending deformation law of the leaf surface of the soft gripper was studied, and a strawberry grasping experiment was conducted using an arcs gas channel soft gripper. The grasping damage rate was 2%, indicating that the developed four-blade soft gripper has good safety when used for strawberry grasping. The pneumatic gripper designed here was modeled according to the external contours of the grasping object to ensure that the outer surface of the grasping object is completely wrapped and protected, which lays a foundation for the further application of the robot pneumatic gripper.

**Figure 14:**
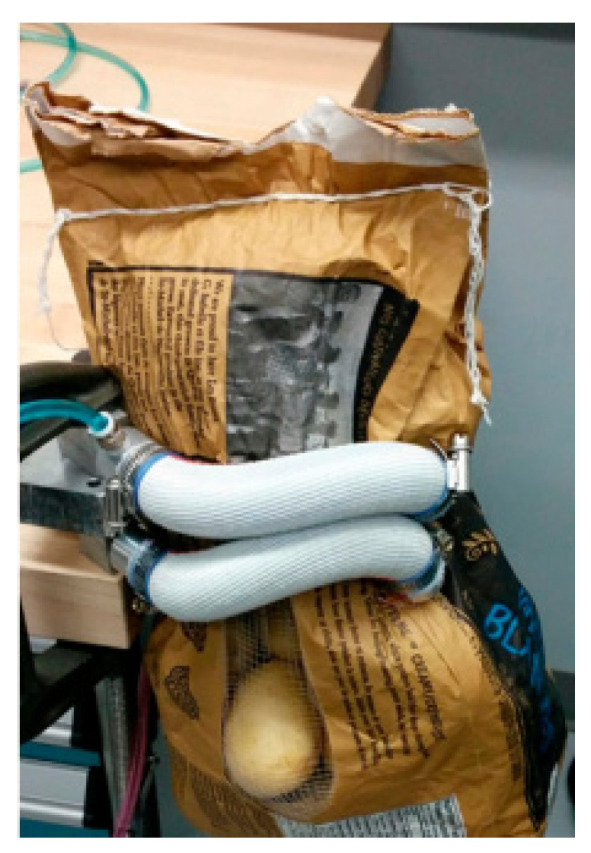
Pneumatic gripper with new sleeve bending fingers [34].

Pneumatic grippers require significant deformation to provide sufficient gripping force when grasping objects, which can limit their durability. Due to the compromise between gripping power and durability, most pneumatic grippers have a maximum load of less than 1 kg. Based on this, Miron et al. [34] proposed a novel sleeve bending finger for pneumatic grippers. The gripper finger sleeve uses silicone tubing as a diaphragm, nylon fabric as a strain limiting layer, and an elastic band to suppress radial expansion while allowing for external longitudinal stretching of the membrane. Compared with the durability of the Kevlar fiber-reinforced gripper fingers currently used, the new sleeve finger has better durability. The sleeve provides a large contact area and minimizes membrane indentation under pressure, thereby limiting strain concentration and surface damage. In addition, the sleeve can withstand greater pressure than individual polymers, and it also serves as a protective film to prevent sharp objects from the environment from piercing the load. The pneumatic gripper is shown in Figure 14; it can grip 20 kg of items with low damage and significantly improves durability.

**Figure 15:**
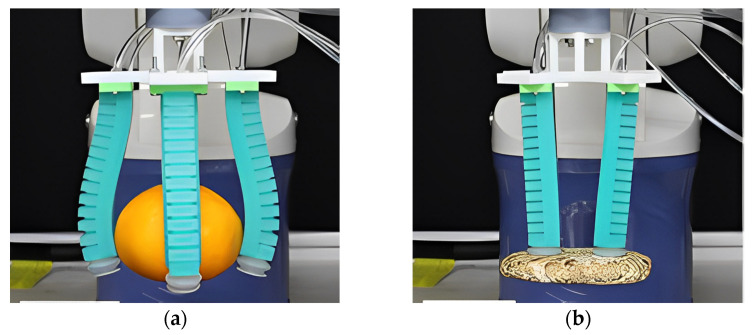
Dual-mode pneumatic soft gripper with gripping and adsorption functions: (**a**) The fingers arranged vertically with each other can be inflated and deflated to achieve the grip function [35]. (**b**) The vacuum suction cup at the end of the finger can achieve adsorption function [35].

Most types of grippers usually operate via one type of actuation method. In order to integrate the benefits of different actuation methods, hybrid actuation grippers are gradually being developed and applied to achieve high performance. Wang et al. [35] proposed a dual-mode soft pneumatic gripper made of rubber material, which can grip and suck on different types of objects. The gripper consists of four soft fingers, each made of rubber material and constructed through casting technology, with a suction cup integrated at the tip of each finger. When designing the finger, they introduced a new gas-path design, eliminated the “strain limiting layer” of the PneuNets actuator, and designed a gas-path layer to allow grabbing and adsorption to co-occur, and then performed finite element method simulations to confirm the finger design. As shown in Figure 15a, the fingers arranged vertically with each other can be inflated and deflated to achieve the grip function. As shown in Figure 15b, the vacuum suckers at the ends of the fingers can achieve the adsorption function.

### 2.3. Vacuum Grippers

Vacuum suction is one of the oldest gripping methods, and can generate adhesion through the negative pressure induced at the interface between the suction cup and the object [36]. The suction force is relatively large and can be used to grip small, large, and heavy products with minimal damage to the product. After pushing the vacuum gripper onto the surface of the object, the pushing force forces the internal air to be expelled, reducing the internal pressure to below atmospheric pressure. The resulting pressure difference causes a vacuum to form between the object and the gripper, resulting in vacuum grasping. Alternatively, one can use a pump or flow generator (such as a Venturi injector) to create a pressure vacuum to expel the air between the object and the vacuum gripper.

The added value of animal viscera products is high, and there is a shortage of labor in Australia and New Zealand, as well as very little equipment available for visceral sorting. Stommel et al. [37] discussed methods for the automated sorting of animal organs, particularly in relation to sorting organs from sheep carcasses, and proposed a scheme for removing individual organs. In this scheme, one arm of the dual arm robot was equipped with a soft vacuum gripper at the end, which grasped and lifted the target organ, while the other arm was used to cut and separate the connecting soft tissue of the organ.

**Figure 16:**
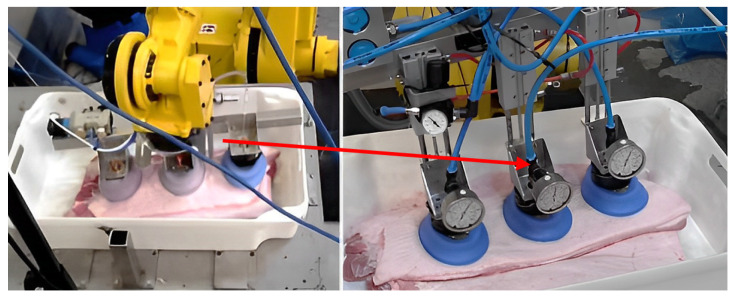
Fully automatic robotic arm with vacuum gripper (6-DOF).

**Figure 17:**
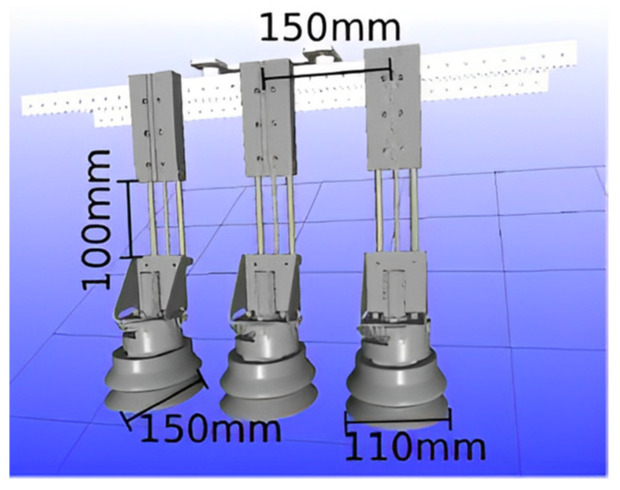
CAD diagram of vacuum gripper.

After the removal of internal organs, the segmentation and processing of the carcass will continue, and the segmented meat pieces still require automated sorting. In order to solve the problem of the automatic grasping and transport of meat pieces, Jørgensen et al. [38] proposed a mechanical arm with six degrees of freedom (6-DOF) and a vacuum gripper based on suction to grasp and lift segmented pork onto the conveyor, as shown in Figure 16. The vacuum gripper consists of three elliptical suction cups, with a suction cup size of 110 mm × 150 mm, installed on the same sliding rod. The grasping experiment showed that the best position of the suckers was the equidistant arrangement, with the cups 150 mm apart. Due to the uneven height of the surface of the meat block, some suckers failed to adsorb successfully during suction. Therefore, the suckers were connected to the spring damping cylinder with a 100 mm stroke, as shown in Figure 17, so that the suckers had enough stroke to adapt to the irregular surface of the meat block. In addition, during the lifting process after grasping, the objects below the meat block often formed a vacuum and adhered to the grasped meat block, which increased the probability of lifting failure. As a result, he proposed two more methods of safe grasping to minimize the impact of this phenomenon. Both of these grasping methods were based on artificial intelligence, using segmented point clouds on the surface of the meat block to determine the clamping motion trajectory [39]. The generation of point clouds was achieved through a 3D visual system. One method is called the “flat grasp method”, which is based on the principal component analysis of point cloud data to determine the required height for grasping. When the suction cup was lowered to a height of 40 mm from the meat block, the vacuum was opened for adsorption and grasping. Another method, called “rolling grasp method”, used point cloud data and the PCL concave shell algorithm to analyze the range of meat block edges that were allowed to be adsorbed by the suction cup, and controlled the suction cup to remain within the meat block edges [40]. Then, a rolling lifting suction was performed, ultimately eliminating the adverse effects of vacuum and adhesion caused by the formation. Experiments have verified that the “rolling grasp method” is the optimal grasp method.

Due to the fact that non-contact vacuum grippers do not come into contact with the surface of the object during grasping, they have a better effect on reducing damage to the object. Therefore, researchers have conducted extensive research on non-contact vacuum grippers made using the Bernoulli principle.

**Figure 18:**
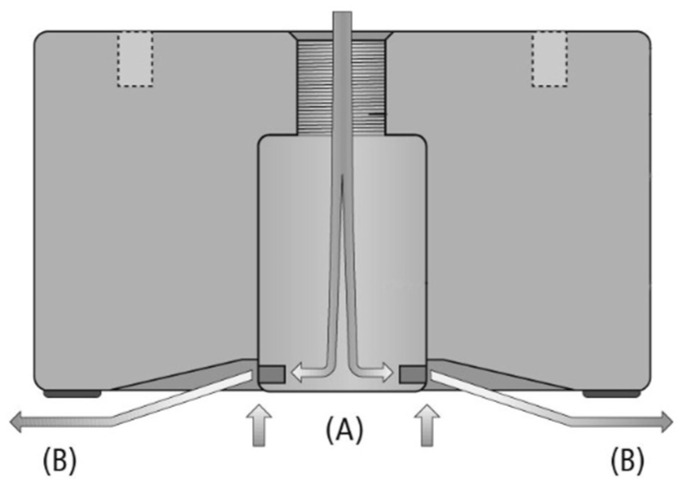
Principle diagram of Bernoulli effect on object grasping: Due to the increase in speed, the static pressure falls and a vacuum is produced (A); The accelerated air escapes to the side (B) [41].

The Bernoulli principle indicates that as the flow velocity of a fluid increases, its pressure decreases. Due to the higher velocity of airflow above the wing of an aircraft, the pressure above the wing is less than that below the wing, resulting in a net upward force. The Bernoulli principle is applied to the lifting force of objects, which is consistent with the lifting force on the wing of an aircraft. Figure 18 shows how to use Bernoulli’s principle to grasp objects [41]. Firstly, compressed air escapes and accelerates through the holes on the suction cup, resulting in a decrease in static air pressure and the creation of a vacuum at Figure 18A. Then, the accelerated air escapes to the side shown in Figure 18B, causing the air to pass through the upper surface of the lifted object, thereby reducing the air pressure above the object. The pressure difference between the upper and lower surfaces of an object creates attraction between the gripper and the object, thereby permitting grasping of the object, in accordance with Bernoulli’s principle. Although the suction force of Bernoulli grippers is relatively small, they are also widely used for the suction and transportation of lightweight workpieces due to their reliability, durability, low air pressure requirements, and greater economy.

**Figure 19:**
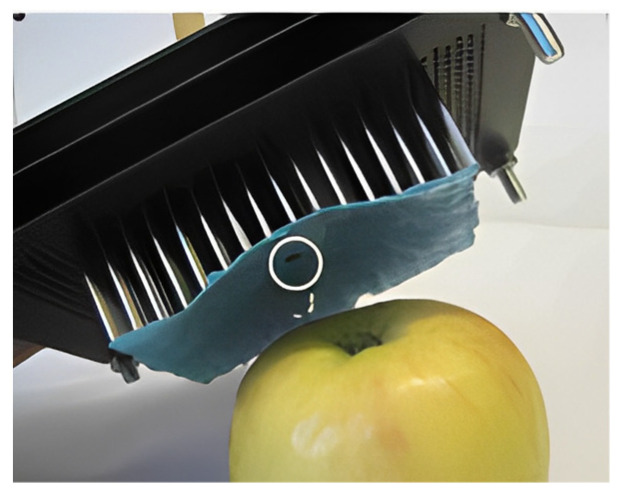
Bernoulli gripper capable of grasping 2D and 3D objects.

Traditionally, Bernoulli grippers have been able to handle a range of different products, which are usually flat and 2D, and they cannot provide sufficient lifting force for most non-flat objects. Petterson et al. [42] designed a novel Bernoulli gripper, as shown in Figure 19. The advantage of this gripper is its ability to grasp 2D and 3D objects. In order to be able to grasp 3D objects, they designed a deformable surface based on a matrix pin board, which includes 16 rows and 21 columns of pins. The surfaces of the pins are covered with a 1.5 mm thin latex rubber plate that is bonded to each pin to form a continuous surface. When grasping, the gripper can be gently pressed onto the top of the target object using a deformable surface. At this point, the surface of the gripper will deform with the contour of the object’s surface, forming an accurate replica. After the molding is completed, the locking mechanism is activated to lock the shape of the grip surface. Then, the gripper can lift a few tenths of a millimeter from the object and form a narrow air gap between the gripper and the surface of the object. By applying airflow to this gap, the static pressure at the top of the object can be reduced, thereby generating lift for object grasping. The deformable surface of the Bernoulli gripper can generate sufficiently low force on the surface of the object being grasped to avoid product damage.

### 2.4. Magnetic Grippers

The shapes of natural foods are usually different, which can easily lead to damage during the grasping process, making it difficult for conventional robots to handle them [43]. In recent years, researchers have developed a class of new materials for soft gripper grasping [44], which mainly include magneto-rheological (MR) fluid [45], MR elastomer [46], MR gel [47] and MR foam [48]. The basic principles of their grasping are the same. Taking the working principle of MR fluid as an example, MR fluid is a functional fluid that can change viscosity under the action of a magnetic field. It is mainly composed of oil and 5–10 µm iron particles, and also includes surfactants for diffusion and preventing the iron powder from settling in the oil. During the working process, applying a magnetic field increases the viscosity of the MR fluid by 105 to 106 times, and the response is very fast, at less than 1 ms [49]. When there is no magnetic field, the iron particles are suspended in the oil, and the MR fluid behaves as a simple fluid; when a magnetic field exists, the iron particles aggregate along the magnetic flux line and form a stable chain-like structure [50].

**Figure 20:**
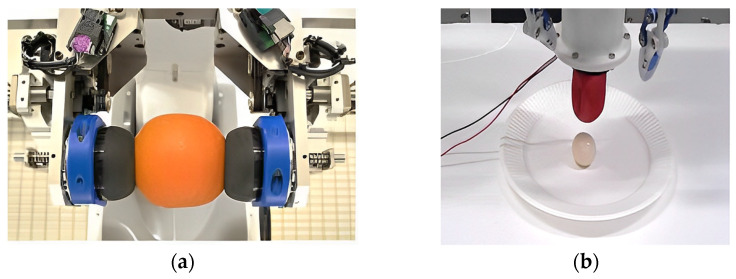
Magnetic gripper: (**a**) Universal parallel gripper using improved MR fluid. (**b**) Grip composed of electromagnet, permanent magnet, elastic film and water.

During the activation of the magnetic field, the MR fluid undergoes morphological solidification for gripping. During the gripping process, hard contact can be avoided, and the soft grip surface is also conducive to handling various shapes and fragile foods. Tsugami et al. [51] developed a universal parallel gripper using MR fluid, as shown in Figure 20a. The gripper consists of two fingers made of elastic membranes filled with MR fluid. When grasping, the MR fluid in the elastic film was not activated at first, but maintained a flowing state. Then, the gripper clamped the target object. In this process, the soft elastic film wrapped the object without producing a clamping force on the object, such that the elastic film maintained the same external profile as the object. Finally, the MR fluid in the elastic film was activated to solidify the outer contour of the elastic film, so as to keep the object from falling off in the elastic mode, enabling it to achieve an effect similar to clamping. After releasing the object, the magnetic field was removed and the deformed elastic film of the gripper quickly returned to its original shape. This gripper could quickly grasp soft and fragile objects with irregular shapes, and its grasping performance was tested in experiments using oranges and other fragile objects.

Tsugami and Nishida [52] also proposed another type of gripper with a simple structure, which is composed of an electromagnet, a permanent magnet, an elastic film and water. The process of grasping and releasing can be divided into the following steps: the gripper pressed onto the target object and covered it with an elastic film → the gripper continued to move downwards → the electromagnet adsorbed the permanent magnet → the elastic film adapted to the shape of the object → the frictional force generated on the contact surface between the object and the elastic film enabled the gripper to grasp the object. As shown in Figure 20b, boiled quail eggs were used for the grasping experiment, and the results show that the proposed gripper could successfully grasp and transport objects without damaging them. The gripper has the advantages of a simple design, low cost, light weight and robustness to misalignments in object grasping.

Based on the MR fluid technology, Pettersson et al. [43] proposed a novel general robot soft gripper, which is used for grasping operations in the food industry and realizes the grasping of food raw materials of various shapes and sizes with little force. He established a robot workstation and conducted grasping experiments on fresh fruits and vegetables such as carrots, strawberries, apples, tomatoes, grapes and broccoli. He used a visual system to extract contour data of the captured object for grasping control. The experimental results show that the robot’s soft gripper can handle various food raw materials without the need for time-consuming readjustments between different food ingredients and without causing damage to food raw materials.

The gripping performance of MR fluid grippers depends on the MR material, so the preparation of MR material is particularly crucial. Guan et al. [53] developed a new hybrid MR material, in which MR fluid is encapsulated in the MR elastomer, and the hybrid MR material reaches a higher MR fluid performance and higher structural stability in the MR elastomer. This hybrid MR material was prepared through the 3D printing method for use in robot soft grippers. The reversibility, stability and grip of the 3D-printed robot soft gripper can be precisely controlled by adjusting the magnetic field. This work not only proposes a new method for manufacturing high-performance and complex 3D hybrid MR materials, but also produces a new type of robot soft gripper that can be used for grasping various external contour objects.

## 3. Research Progress of Control Strategies for Grasping

The reason why human hands can grasp most objects is not only because of their flexible joints, but also because they can be seen as a composite grasping system that integrates sensors and handling tools. Under the control of the brain, human hands can provide stable, adaptable, flexible grasping, and other excellent operational abilities, through sensory feedback and predictive knowledge. Robots also need to interact with various sensors during grasping, including visual sensors, tactile sensors, motion sensors and distance sensors, to interact with variable and complex environments [54], and automatically develop and implement closed-loop control strategies [55,56]. Robotic grippers attempt to imitate the advantageous aptitudes of human hands by using multiple sensors in grasping various kinds of products, exploiting the senses of touch and visual perception [26]. By integrating multiple sensors, the gripper can not only serve as the end tool of the robot, allowing it to grasp objects, but the information between the gripper and the object can also be analyzed, making the gripper more intelligent.

### 3.1. Control Strategy Based on Visual Sensors

Visual sensors are devices that convert optical images into electronic signals. They are the most suitable non-contact sensors for robots to obtain information, and are commonly used to locate objects, determine grip points in the workspace, and identify surrounding obstacles. Visual sensors are also used to detect contact, measure the three-dimensional structure of objects, and provide visual feedback by tracking the positional relationship between the gripper and the captured object [57]. Therefore, integrating visual sensors into the gripper or robot can effectively control motion and the grasping process.

**Figure 21:**
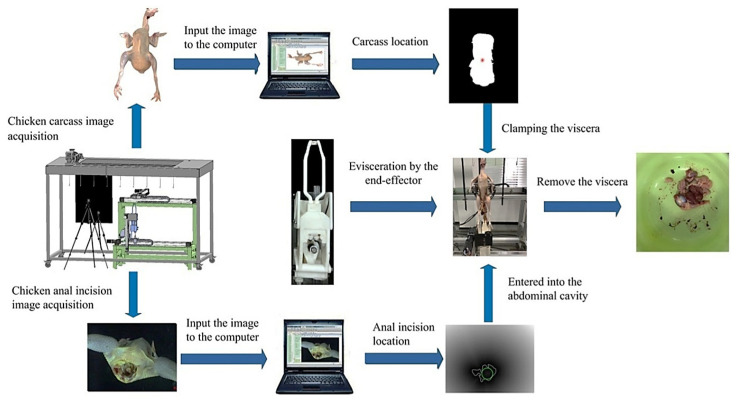
Working process of a chicken automatic visceral removal robot system based on machine vision [58].

Automatic evisceration technology has been used in large-scale poultry slaughtering and processing enterprises. Compared with manual evisceration, automatic evisceration technology has many obvious application advantages, such as lower labor intensity, higher production efficiency, and a better working environment. At present, many of the design concepts for automatic visceral removal systems with different structures are similar to each other. Usually, the chicken is placed on a synchronous conveyor, and the end effector is controlled by a spatial cam mechanism, enabling it to complete visceral operations. However, these visceral robotic arms with singular fixed structures have been proven to easily damage internal organs, including the liver and intestines, and general, automatic evisceration equipment may not be suitable for chickens of different sizes. Robot evisceration devices can provide potential means to reduce the rate of visceral damage and meet the size consistency requirements in chicken processing. In recent years, researchers have conducted further research on automatic emptying robots based on machine vision technology. Chen et al. [58] developed a robot system for automatic chicken viscera removal based on machine vision, which was composed of a chicken carcass conveying device, a manipulator, an end effector, a control component and a machine vision component, and its working process is shown in Figure 21. The image of the chicken carcass is collected by the machine vision components, and is then processed by color space conversion, threshold segmentation, morphological operations and the active contour algorithm. The anal contour of the chicken carcass is extracted by the maximum inscribed circle algorithm, and its spatial position is detected to guide the robot to excise the chicken carcass viscera from the anus. The automatic evisceration robot system can successfully complete the evisceration operation in chickens. In summary, the visceral removal method based on machine vision is sufficient to meet the technical requirements of robot visceral removal, and is expected to be successfully applied in robot visceral removal in the poultry industry.

Robot grasping is a key step in completing tasks such as transportation, sorting, and assembly. Currently, the accuracy of robot grasping is influenced by many factors such as unknown object information, grasping planning and design, and the dynamic environment, resulting in a lack of a capacity for the autonomous grasping of unknown objects to meet application requirements. Studying efficient, accurate, and reusable grasping methods is of great significance for improving the efficiency of robotic arms and reducing production costs. In response to the difficulty of robots effectively grasping unknown objects, Cheng et al. [59] proposed a reliable grasping algorithm based on a depth camera and ABB’s dual arm robot system in an unstructured environment. This algorithm used an iterative nearest point coordinate transformation method to first calibrate the position relationship between the camera and the robot. Then, the collected visual depth information was filtered out based on the threshold of gradient size changes to select feature pixels, and candidate points for grabbing were generated. Finally, an improved grasping quality judgment network algorithm was used to obtain the grasping points that could obtain the optimal grasping quality and robot posture. The grasping experiment verified that the algorithm can detect the best grasping points of different objects. The improved algorithm for grasping point quality judgment using deep neural networks proposed by him can effectively solve the problem of the difficulty of effectively grasping unknown objects for robots, which is of great significance in improving the accuracy of robot grasping.

### 3.2. Control Strategy Based on Tactile Sensors

Tactile sensors have a development history of 40 to 50 years, and researchers have been committed to developing tactile systems that simulate humans [60,61]. Tactile sensors have broad application prospects in intelligent fields such as robotic arms, medical rehabilitation and robot systems [62,63,64]. In the field of intelligent robots, tactile sensors feedback the acquired tactile information to the system, which controls the grasping force of the robotic arm, detects the weight of the grasped object, and adjusts the grasping force and direction of the robotic arm based on the downward sliding shear force and squeezing pressure of the object [65,66]. The tactile information obtained by tactile sensors can also be used to supplement the visual system [67,68], thereby achieving stable grasping. Recently, researchers have conducted extensive research on tactile sensors.

Magnetostrictive materials are materials that have the function of converting electromagnetic energy into mechanical energy. By utilizing the inverse magnetostrictive effects of materials, tactile sensors with simple structures, fast reaction speeds, and excellent dynamic characteristics can be designed. Installing the sensor on the robotic arm can allow the used to obtain information on the grasping force and object stiffness of the robotic arm during the grasping process. Inspired by the sensing and detection functions of human fingers, Wang et al. [69] designed a tactile sensor array for measuring pressure and stiffness based on the inverse magnetostrictive effect of the new magnetostrictive material Fa_83_Ga_17_. A model of the tactile sensor array was established, and a two-by-two sensor array prototype was prepared, with the size of 37 × 22 × 14 mm. The sensor array prototype was installed on a two-finger robotic arm for output characteristic testing. The test results show that the sensitivity of the sensor array was 115 mV/N, the detection range was 0–3 N, and it had a fast reaction speed within the period of 0–1 s. The sensor array has a small volume, high sensitivity, and fast response speed, and it can detect tactile pressure and perceive pressure distribution and object stiffness information. The application of magnetostrictive tactile sensor arrays in robotic hand grasping not only increases the additional load capacity of the manipulator, but also helps the manipulator to complete stable grasping tasks.

In recent years, deep learning has shown unique advantages and potential in feature extraction and pattern recognition. In the field of robot tactile research, tactile recognition algorithms based on deep learning have unique advantages, which can enable the complex work of feature extraction to be overcome in traditional methods. The concept of deep learning originated from artificial neural networks, and deep learning models contain multi-layer perceptrons with excellent feature learning capabilities. In deep learning models, the convolutional neural network (CNN) is a multi-layer neural network composed of alternating convolutional and pooling layers, which is suitable for extracting spatial features of data. The long–short-term memory (LSTM) neural network is a time recursive neural network suitable for processing and predicting important events with relatively long intervals and delays in time series. In the time series analysis and recognition of robot gripper tactile arrays, it is not only necessary to consider the spatial relationship of the array data, but also the temporal variation pattern of the data. In addition, many existing single neural network algorithms are often limited in terms of computational speed or accuracy when applied to more complex problems. Therefore, combining CNN and LSTM has been a highly sought after deep learning method in the field of deep learning in recent years. In the algorithm combining CNN and LSTM, CNN is suitable for extracting spatial features of data and reducing data dimensions, while LSTM is good at obtaining temporal features of data and has long-term memory functions, making it suitable for processing time series.

Based on this, Hui et al. [70] proposed a tactile recognition neural network model based on CNN and LSTM for sequence recognition in collected tactile signals. In the experiment, they established a tactile recognition database using 14 experimental samples, and conducted recognition tests on these samples for 14 classifications and 4 classifications, achieving recognition accuracy rates of 94.2% and 95.0%, respectively. The online recognition platform built based on this tactile recognition neural network model can be practically applied to the online tactile recognition of objects, and achieve stable grasping control. The CNN-LSTM model they established can extract tactile data features more comprehensively, with higher recognition rates and a good generalization ability. Combined with existing robot grippers, it can achieve online object classification and stable grasping, effectively reducing object deformation during the grasping process and reducing the maximum gripping force of the gripper.

The above researchers’ research on tactile and visual sensors has been applied to accurate position recognition and precise feedback control during robot grasping. However, the performance of the most advanced tactile or visual sensors developed so far still falls far short of human sensing capabilities. In order to make the control of robot grippers more precise and intelligent, there are still many challenges to be overcome in the future.

## 4. Future Development Trends

Without more intelligent robots, modern food production and processing cannot be achieved. Without low-damage robot grippers and control strategies, the high-quality processing of food raw materials cannot be guaranteed. Due to the demand for adaptable and reliable food-sorting robot grippers, various grippers have been developed and applied in food raw materials sorting and processing over the past few decades. Many gripper products have been designed and released by enterprises and universities, and the work done on the development of robot grippers has made significant contributions to the steady and rapid growth of robot automation applications. Despite the continuous development of various sorting robot grippers that can reduce grasping damage, the future of low-damage grasping using sorting robots remains full of challenges. In the future, hybrid grippers combining different actuating types will have good development prospects. Hybrid grippers can integrate the advantages of different types of grippers, and will have a stronger grasping ability compared to single types of grippers, minimizing the risk of damage to the object being grasped and providing stable grip.

At the same time, due to the complex and unstructured environment, the assessment of the grasping of food raw materials by grippers is also a challenging problem. During the grasping process, it is necessary to clarify the size and position of the target object. Integrating multiple sensors to achieve information fusion and precise control of the gripper may be a solution. Integrating various sensors such as pressure sensors, visual sensors, distance sensors, and tactile sensors into the gripper not only improve positioning accuracy, but also ensures low damage when processing food raw materials, enhancing the adaptability of food-sorting robots to complex operating environments.

In addition, it will be challenging to achieve the optimally flexible and safe grasping control of different food raw materials based on sensory data and physical and biological characteristics. For example, when grasping food raw materials, excessive grasping forces may lead to potential damage, while too low a grasping force may cause objects to slip. In addition, when considering the viscosity of food raw materials and their potential impacts on grasping, grasping speed also plays an important role. There are almost no data for robot sorting, but deriving these data is crucial for designing grippers and researching grasping strategies. Therefore, it is becoming increasingly important to conduct in-depth research on the mechanism of grasping damage, establish a dynamic model to explain the relation between the gripper and the food raw material, and study the impacts of different grasping forces and speeds on the contact stress and grasping damage of food raw materials. In the future, it would be best to develop a food raw material classification system that includes quantitative descriptions of surface conditions, hardness, and damage resistance characteristics [71]. It is necessary to establish a relationship between robot grippers and food categories to help select suitable robot grippers. It would be useful to develop a database containing information on the physical properties and weights of food raw materials related to robot handling, such as viscoelasticity or rheology, friction, geometric shape, and weight.

Finally, considering the cost issue, the versatility of the sorting robot should be improved. Taking into account the common characteristics of similar food raw material sorting approaches, the design of the sorting fixture should be modular, making it suitable for grasping various food raw materials, and thereby improving the versatility of the fixture and reducing the cost of the sorting system.

## Data Availability

The data presented in this study are available on request from the corresponding author.
